# Neuro-functional correlates of personality dimensions in Parkinson’s disease

**DOI:** 10.3389/fphar.2025.1705937

**Published:** 2025-11-07

**Authors:** Mathilde Boussac, Estelle Harroch, Karel Joineau, Emeline Descamps, Christine Brefel-Courbon

**Affiliations:** 1 Toulouse NeuroImaging Center (ToNIC – UMR1214 INSERM/Toulouse University III), Toulouse, France; 2 Department of Clinical Pharmacology and Neurology, University Hospital of Toulouse, Toulouse, France; 3 Centre National de la Recherche Scientifique (CNRS), Toulouse, France

**Keywords:** personality, temperament and character inventory, functional connectivity, rs-fMRI, Parkinson’s disease

## Abstract

**Introduction:**

According to the original model of the Temperament and Character Inventory (TCI), personality dimensions would be related to different neurotransmitters’ systems such as the dopaminergic and the serotoninergic ones.

**Methods:**

Our objective was to study associations between functional connectivity and personality in Parkinson’s disease (PD). The data of 29 PD patients were collected (NCT04705207). It included personality evaluation using the TCI, functional connectivity from resting-state functional MRI, and anxio-depressive state from the Hospital Anxiety and Depression scale (HAD). Seed-to-voxels and ROI-to-ROI analyses were done in the CONN toolbox.

**Results:**

Significant association was found between Novelty Seeking scores and functional connectivity within the nucleus accumbens and one cluster formed of the orbitofrontal cortex. Significant associations were also found between Harm Avoidance scores and functional connectivity within the temporal pole and seven clusters (mainly formed of the post- and pre-central gyri, thalami, parietal lobule, putamen and temporal gyrus). These functional connectivities also correlated with HAD scores.

**Conclusion:**

In accordance with the TCI model, Novelty Seeking seems to be related to the dopaminergic system within the nucleus accumbens and orbitofrontal cortex connectivity, implicated in impulsivity. Moreover, Harm Avoidance would be related to the serotoninergic system within the temporal and fronto-thalamo-parietal network connectivity, involved in depressive disorders.

**Clinical trial registration:**

clinicaltrials.gov: NCT04705207 (https://clinicaltrials.gov/study/NCT04705207).

## Introduction

Parkinson’s disease (PD) is a neurodegenerative disorder, well-known for its motor and non-motor symptoms (Bloem et al., 2021). PD patients were described with a specific personality: inflexible, cautious, etc. (Poewe et al., 1989; Sands, 1942). More recent studies have partially confirmed this hypothesis of a PD “premorbid personality” with lower Novelty Seeking scores and higher Harm Avoidance scores in comparisons to healthy controls (Menza et al., 1990; Santangelo et al., 2018). Most of these studies were done using the Temperament and Character Inventory (TCI) (Cloninger et al., 1994).

We continued to investigate personality in PD using the TCI (Boussac et al., 2022; Boussac et al., 2022; Boussac et al., 2021; Boussac et al., 2024), and differences were observed between PD patients and a French normative population (Boussac et al., 2021). Our findings also suggested that the personality of PD patients may evolve over the course of the disease, either due to the dopaminergic treatments, the neurodegeneration or others aspects of disease progression (Boussac et al., 2021). For instance, PD patients awaiting deep brain stimulation of the sub-thalamic nucleus (DBS-STN) or continuous subcutaneous apomorphine infusion, may exhibit a “specific” personality, characterized by higher scores in some dimensions compared to *De Novo* PD patients (Boussac et al., 2021; Boussac et al., 2024; Meira et al., 2022). The progression of PD, with the emergence of motor fluctuations, or the psychological burden associated with decision-making concerning invasive therapies, or the stress of awaiting a second-line treatment could explain these findings (Boussac et al., 2021). Dopaminergic treatments may also affect personality dimensions as they are related to certain neurotransmitters systems (Cloninger et al., 1994), although no correlation has been found between the dose of dopaminergic treatments and personality in PD (Boussac et al., 2022).

The seven personality dimensions of the TCI are divided into four temperaments and three characters, according to R. Cloninger original model (Cloninger et al., 1994). In this model, the temperaments are personality dimensions supposedly related to different part of the brain and neurotransmitter’s systems: the Novelty Seeking would be associated with the dopaminergic system, the Harm Avoidance with the serotoninergic system, the Reward Dependence with the noradrenergic system, and the Persistence with the glutamatergic one (Hansenne, 2001). Nonetheless, studies are lacking to confirm these relationships. At the beginning, mainly genetic studies were done to validate these hypotheses and some correlations between these temperament scores and different polymorphisms were found (Benjamin et al., 1996; Ham et al., 2005; Ricketts et al., 1998). Then, imaging studies mostly evaluated the volume of different brain regions using voxel-based morphometry in healthy subjects (Gardini et al., 2009; Iidaka et al., 2006; Laricchiuta et al., 2012; Laricchiuta et al., 2013; Petrosini et al., 2015; Petrosini et al., 2017; Picerni et al., 2013; Van Schuerbeek et al., 2011), and results differed according to the studies, except concerning the cerebellum volumes which correlated positively with Novelty Seeking scores, and negatively with Harm Avoidance (Laricchiuta et al., 2012; Petrosini et al., 2015; Petrosini et al., 2017; Picerni et al., 2013). Some positron emission tomography studies also explored the metabolism (Park et al., 2010), serotoninergic (Tuominen et al., 2013) or dopaminergic (Kaasinen et al., 2001; Kaasinen et al., 2004) systems in relation to personality dimensions, and contradictory results were found. Indeed, the dopaminergic system was either related to Novelty Seeking, or to Harm Avoidance (Kaasinen et al., 2001; Kaasinen et al., 2004). Lastly, only two studies used fMRI (Kyeong et al., 2012; Sisara et al., 2024) but none studied functional connectivity.

Therefore, we aimed to further study how personality dimensions are related to brain systems using functional connectivity in PD, an interesting model since personality can change in PD (Boussac et al., 2021), and cerebral systems are affected by this neurodegenerative disease (Buddhala et al., 2015). Hence, we used imaging data from an already published study, DOREPAR, in PD patients (Descamps et al., 2023). Our objective was to evaluate associations between TCI personality dimensions scores and brain functional connectivity in PD. Based on the literature, we proposed that TCI temperaments would be associated with functional connectivity within networks or regions involved in the dopaminergic, serotoninergic, noradrenergic or even glutamatergic systems (Cloninger et al., 1994; Hansenne, 2001; Pélissolo and Lépine, 2000). Indeed, we hypothesized that Novelty Seeking scores would be related to different regions involved in the reward system (ventral tegmental area, nucleus accumbens etc.), which are also implicated in impulsive behaviour (Flores-Dourojeanni et al., 2021; Shen et al., 2024). Harm Avoidance scores would be associated with the amygdala, the hippocampus, and the temporal regions, linked to the serotoninergic system and depressive disorders (Newberg et al., 2012; Wang et al., 2016). Reward Dependence scores would be correlated to frontal regions and to the locus coeruleus, where noradrenaline is produced (Bari et al., 2019). Concerning the Persistence temperament, we had fewer ‘*a priori*’ assumptions concerning its association with the glutamatergic system, since it is the more recently added temperament in the TCI model, comprising only eight questions, therefore lacking strong validation (Cloninger et al., 1994).

## Methods

### Study design and patients

This is an ancillary study of the DOREPAR study (clinicaltrials.gov: NCT04705207), approved by the “CPP Sud-Ouest et Outre-Mer III” ethical committee (n°2020-A03036-33). Complete description of the study design can be found in Karel et al. (2025) and Joineau et al. (2025).

Idiopathic PD patients (according to the UKPDSBB), aged 18 years and older, without disabling dyskinesias and experiencing chronic pain were included. Patients with cognitive impairments (Montreal Cognitive Assessment score (MoCA) < 25), and with contraindications to MRI, including claustrophobia, were excluded.

Demographical and clinical data (gender, age, disease duration, levodopa equivalent dosage (LED) (Tomlinson et al., 2010) etc.) were collected. Anxio-depressive state was measured with the Hospital Anxiety and Depression scale (HAD), and personality dimensions were assessed with the TCI. The TCI is an auto-evaluation formed of 226 True or False questions, which results in seven personality dimensions scores (Cloninger et al., 1994). Then, resting-state functional magnetic resonance imaging (rs-fMRI) evaluated brain functional connectivity. Patients were under their usual dopaminergic treatment throughout the whole protocol.

This study was carried out in accordance with the Declaration of Helsinki. Every patient gave their written and oral consent. Their rights to privacy were observed throughout the study.

### Imaging acquisition and processing

MRI images were acquired on a Philips 3T, and included structural (T1) and rs-fMRI images. For T1 images: MPRAGE sequences were conducted with a 3D sagittal acquisition, square FOV = 240 mm, 1 × 1 × 1 mm^3^, TR = 7.5 ms, TI = 900 ms, TE = 3.5 ms, flip angle = 8°, no fat suppression, full k-space, no averages. For functional images: EPI sequences were conducted with a nominal voxel size of 3 × 3 × 3 mm^3^, TR = 2.0s, TE = 30 ms, α = 90° (Ernst angle), equidistant interleaved slice order, 3 mm slice gap, 41 slices, 300 volumes, and no parallel imaging. Acquisition time was 10 min. Participants were instructed to relax, keep their eyes closed and try to avoid thinking during examination.

Data were processed with the CONN toolbox version 21.a (Whitfield-Gabrieli and Nieto-Castanon, 2012) with the same method as Joineau et al. (2024). First, functional data were realigned: all scans were co-registered and resampled to a reference image. Then, temporal misalignments between different slices were corrected. Outlier scans were identified based on the global Blood Oxygen Level Dependent (BOLD) signal and subject motion in the scanner, and these outlier scans were excluded from the dataset. Following this, both functional and anatomical data were normalized into standard Montreal Neurological Institute (MNI) space and segmented into grey matter, white matter, and cerebral spinal fluid. The functional data underwent smoothing through spatial convolution using a Gaussian kernel with a full-width half-maximum of 8 mm. After smoothing, potential confounding effects on the estimated BOLD signal were estimated and removed separately for each voxel, each subject, and functional run using the aCompCor method. Finally, the residual BOLD time series underwent band-pass filtering within a low-frequency window of interest (0.009 Hz < frequency <0.08 Hz). Quality control plots were analyzed to assess the denoising process before proceeding to the first-level analysis.

In Seed-to-Voxel analyses, different regions of interest (ROIs) were chosen as seeds according to the literature (Hansenne, 2001), mainly to the description of R. Cloninger of its TCI personality model (Cloninger et al., 1994). Different ROIs were selected for the different temperaments of personality evaluated, according to the hypothesized neurotransmitter system they were supposedly related to. We selected the: ventral tegmental area (VTA), left and right nucleus accumbens, and left and right orbitofrontal cortex for the Novelty Seeking temperament related to the dopaminergic system; left and right amygdala, left and right temporal poles, and left and right hippocampus for the Harm Avoidance temperament related to the serotoninergic system; VTA, left and right frontal poles, and brain stem for the Reward Dependence temperament related to the noradrenergic system; and left and right hippocampus, and left and right nucleus accumbens for the Persistence temperament related to the glutamatergic system. The Harvard-Oxford atlas, implemented in CONN, was used to segment these ROIs. In Seed-to-Voxels analyses, only the four temperaments of personality were evaluated to assess their associations with functional connectivity, since they are the only dimensions with hypotheses of cerebral relationships, contrary to the characters.

In the first-level analyses, Pearson’s coefficients were calculated between the seed’s time course and the time courses of all other voxels in the brain, and then transformed into normally distributed scores using Fischer transformation. Then, a general linear model was computed for testing statistical hypotheses in the second-level analyses. For clustering, the Gaussian Random Field theory method was applied with a cluster threshold corrected for multiple comparisons (p < 0.05).

In ROI-to-ROI analyses, we have chosen to explore the functional connectivity in several network implicated in the sensory and attentional processes: the Default Mode Network (DMN), the Sensory Motor Network (SMN), the Executive Control Network (ECN) and the Salience Network (SN), implemented in CONN. Here, the seven personality dimensions were evaluated to study their associations with functional connectivity, since there are no hypotheses concerning cerebral network and personality.

### Statistical analyses

The demographical and clinical data were described using mean and standard deviation, and headcount and percentages for the categorical variables.

For our main objective using Seed-to-Voxel analyses, we performed linear regressions between TCI scores (separately for the four temperaments) and functional connectivity measures. Concerning the ROI-to-ROI analyses, we performed linear regressions between TCI scores (separately for the seven personality dimensions) and functional connectivity measures. FDR-corrected p.values, directly implemented in CONN modeling, were extracting and used to evaluate significant associations between TCI dimensions scores and functional connectivity measures.

Then, Pearson correlations were done between significant clusters of functional connectivity measures and relevant clinical variables (TCI temperaments, LED, HAD scores, age, disease duration, mean VAS of pain, MoCA scores, and part III of the MDS-UPDRS (Movement Disorders Society-Unified Parkinsons’s Disease Rating Scale)).

Statistical description of clinical variables was performed on RStudio (version 2024.12.1). All statistical analyses involving connectivity were performed on CONN, and data of significant clusters were then exported in RStudio to confirm their correlations with TCI scores, and to perform the correlations with clinical variables. FDR-corrected p.values < 0.05 were considered as significant for the rs-fMRI analyses. For the correlation analyses, we used Bonferroni to correct for multiple comparisons leading to a threshold of significativity of p.value < 0.0038 (for 13 comparisons).

## Results

Description of the population is presented in Table 1. PD patients (n = 29), mainly women (59%), had a mean age of 66.0 ± 7.5 years old and a mean disease duration of 6.9 ± 4.7 years.

**TABLE 1:** Description of the population (n = 29).

Variables	Population (n = 29)		
	Mean ± sd	Min	Max
Age (years)	66.0 ± 7.5	52	80
Sex (F; M)	17 ± 12	-	-
Disease duration (years)	6.9 ± 4.7	1	21
Pain duration (years)	9 ± 8.84	1	41
Mean VAS	55.31 ± 14.1	30	80
MoCA	27.97 ± 1.82	24	30
LED (mg/day)	745.98 ± 407.66	120	1,625
MDS-UPDRS part III	25.07 ± 16.06	4	64
HAD total	16.29 ± 6.07	3	27
HAD anxiety	9.54 ± 3.75	3	15
HAD depression	6.75 ± 3.68	0	13
Temperament and character inventory			
Novelty seeking	17.21 ± 4.95	7	27
Harm avoidance	20.1 ± 7.04	3	35
Reward dependence	15.07 ± 3.83	7	21
Persistance	5.55 ± 1.68	3	8
Self-directedness	32.76 ± 5.44	19	41
Cooperativeness	34 ± 3.63	24	40
Self-transcendance	15.69 ± 6.84	4	31

Values are given in mean ± standard deviation and range. VAS, visual analog scale; MoCA, montreal cognitive assessment; LED, levodopa equivalent dosage; MDS-UPDRS, Movement Disorder Society-Unified Parkinson’s Disease Rating Scale; HAD, hospital anxiety disorder scale.

### Seed-to-voxel analyses

**FIGURE 1:**
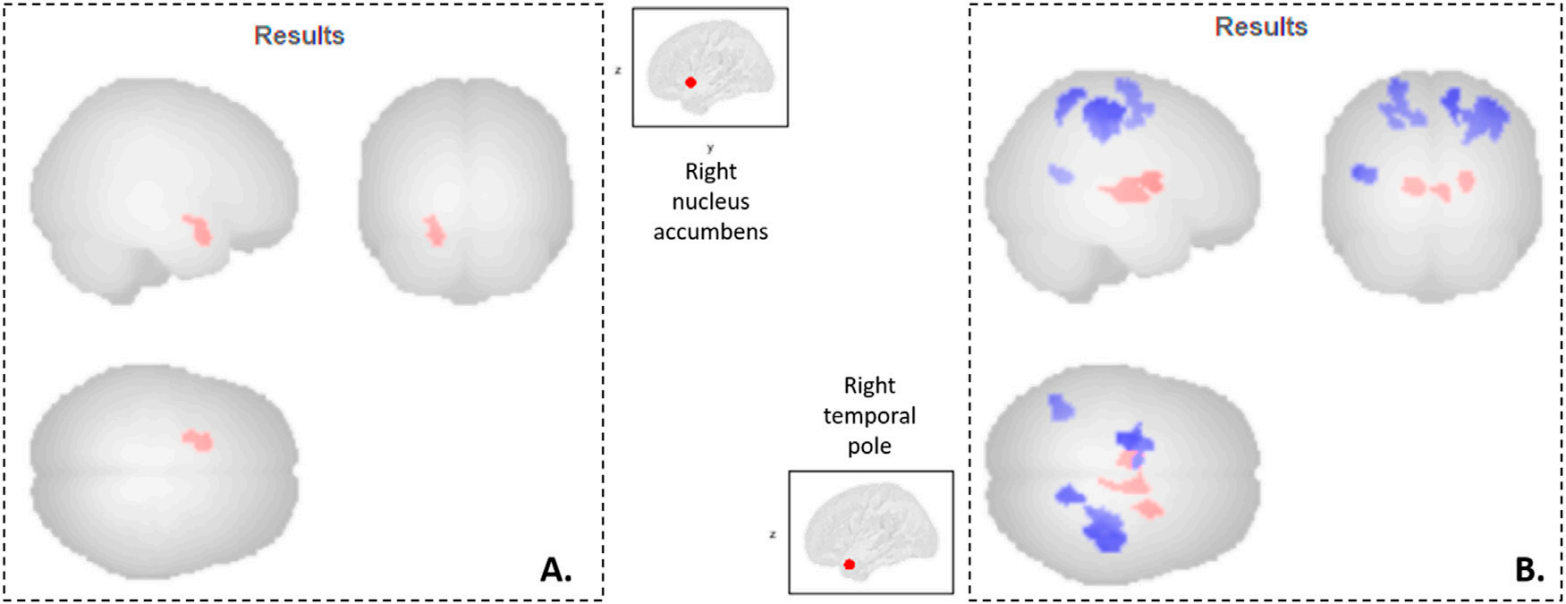
Neuro-functional correlates of the Temperament and Character Inventory in Parkinson’s disease patients. Seed-to-voxel analyses: **(A)** Cluster 1 *(-18 +06–22, 146 voxels, p-FDR = 0.02)* mainly formed by the left orbitofrontal cortex, whom functional connectivity with the right nucleus accumbens is associated with TCI Novelty Seeking scores; **(B)** Clusters mainly formed by the right post-central cortex *(cluster 2: +38–28 +50, 743 voxels, p-FDR = 10* ^*–6*^ *)*, the left pre-central cortex *(cluster 3: -22–12 +68, 324 voxels, p-FDR = 0.0004)*, the right thalamus *(cluster 4: +10–08 +00, 177 voxels, p-FDR = 0.008)*, the left thalamus *(cluster 5: -08–12 +04, 174 voxels, p-FDR = 0.008)*, the right superior parietal lobe *(cluster 6: +14–52 +62, 141 voxels, p-FDR = 0.02)*, the right putamen *(cluster 7: +22 +00 +12, 133 voxels, p-FDR = 0.02)*, and the left middle temporal gyrus *(cluster 8: -38–58 +12, 131 voxels, p-FDR = 0.02)*, whom functional connectivity with the right temporal pole is associated with TCI Harm Avoidance scores. In red, functional connectivity is positively associated with TCI scores; while in blue, it is negatively associated with TCI scores.

Two significant results were observed (Figure 1).

**FIGURE 2:**
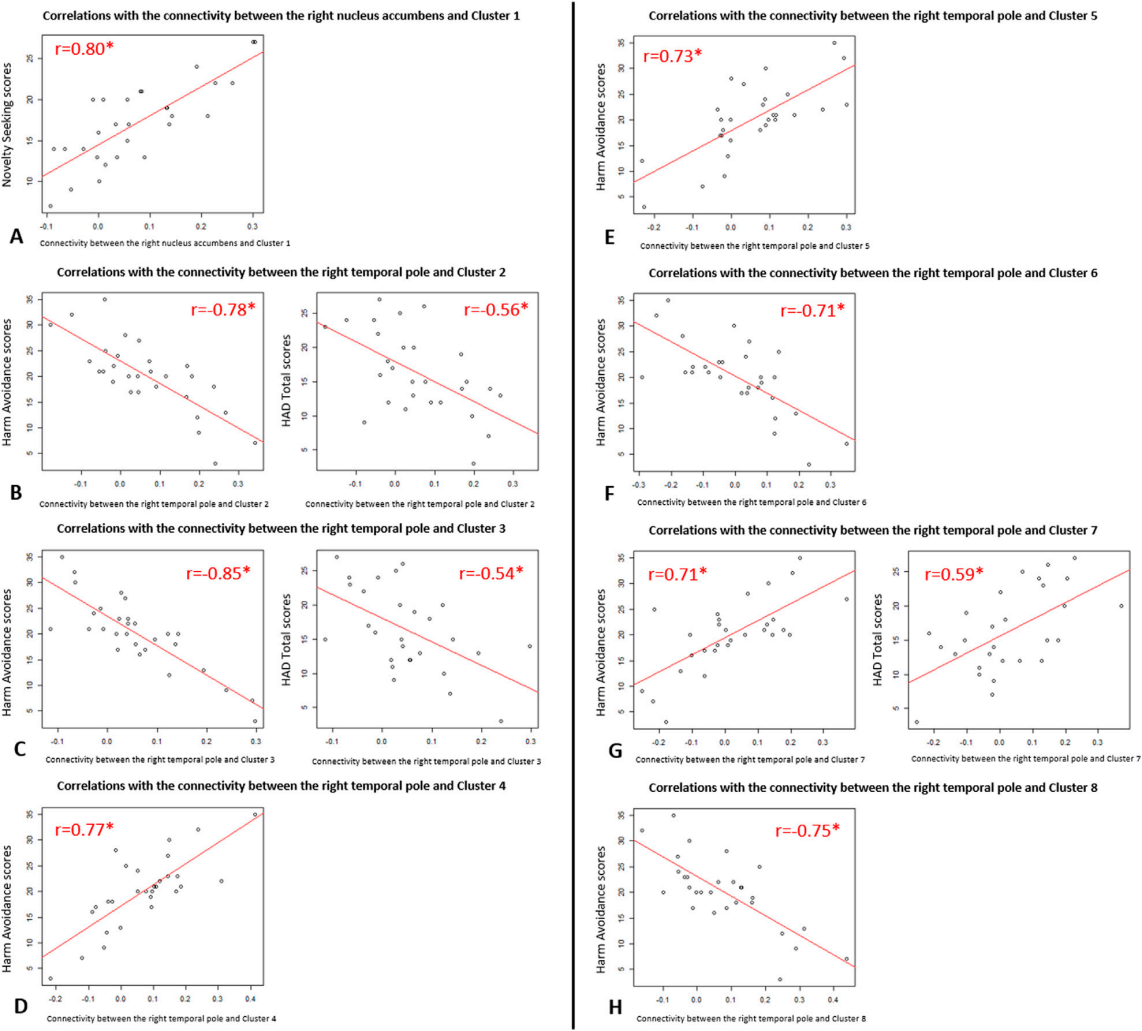
Scatter plots of the significant correlations between functional connectivity and clinical variables in Parkinson’s disease patients. Representations of the correlations of Pearson from Table 2: **(A)** Correlations between the functional connectivity of “the right nucleus accumbens and Cluster 1” and Novelty Seeking scores; **(B)** Correlations between the functional connectivity of “the right temporal pole and Cluster 2” and Harm Avoidance scores (on the left) and HAD Total scores (on the right); **(C)** Correlations between the functional connectivity of “the right temporal pole and Cluster 3” and Harm Avoidance scores (on the left) and HAD Total scores (on the right); **(D)** Correlations between the functional connectivity of “the right temporal pole and Cluster 4” and Harm Avoidance scores; **(E)** Correlations between the functional connectivity of “the right temporal pole and Cluster 5” and Harm Avoidance scores; **(F)** Correlations between the functional connectivity of “the right temporal pole and Cluster 6” and Harm Avoidance scores; **(G)** Correlations between the functional connectivity of “the right temporal pole and Cluster 7” and Harm Avoidance scores (on the left) and HAD Total scores (on the right); **(H)** Correlations between the functional connectivity of “the right temporal pole and Cluster 8” and Harm Avoidance scores.

There was a significant positive correlation between Novelty Seeking scores and the functional connectivity between the right nucleus accumbens and one cluster *(cluster 1)* mainly formed of the left orbitofrontal cortex (Figure 1A). Exportation of connectivity values within this cluster confirmed the positive and significant correlation with Novelty Seeking scores (Table 2 and Figure 2). Nonetheless, this functional connectivity between the right nucleus accumbens and cluster 1 did not correlate with LED, neither with the other variables evaluated (Supplementary Table S1).

**TABLE 2:** Correlations between TCI dimensions and functional connectivity in Seed-to-Voxels analyses.

TCI dimensions	Seeds	Clusters	Coordinates	Coefficients of correlation with TCI^#^	Coefficients of correlations with HAD total^$^
Novelty seeking	Right nucleus accumbens	Cluster 1	*−18 + 06–22*	0.80*	-
Harm avoidance	Right temporal pole	Cluster 2	*+38–28 +50*	−0.78*	−0.56*
		Cluster 3	*−22–12 +68*	−0.85*	−0.54*
		Cluster 4	*+10–08 +00*	0.77*	0.47
		Cluster 5	*−08–12 +04*	0.73*	0.36
		Cluster 6	*+14–52 +62*	−0.71*	−0.43
		Cluster 7	*+22 +00 +12*	0.71*	0.59*
		Cluster 8	*−38–58 +12*	−0.75*	−0.47

# Correlations between functional connectivity and TCI, dimensions scores; ^$^ Correlations between functional connectivity and Total HAD, scores; *p.value < 0.0038 correcting for multiple comparisons with Bonferroni.

There were significant correlations between Harm Avoidance scores and the functional connectivity between the right temporal pole and seven clusters, mainly composed of the: right post-central gyrus *(cluster 2)*, left pre-central gyrus *(cluster 3)*, right thalamus *(cluster 4)*, left thalamus *(cluster 5),* right superior parietal lobule *(cluster 6)*, right putamen *(cluster 7),* and left middle temporal gyrus *(cluster 8)* (Figure 1B). Correlations were positive for the connectivity with the clusters 4, 5 and 7, and negative for the clusters 2, 3, 6 and 8. Exportation of connectivity values within these clusters confirmed their significant correlations with Harm Avoidance scores (Table 2 and Figure 2).

Moreover, functional connectivity between the right temporal pole and three of these seven clusters were also significantly correlated with Total HAD scores: correlation positive for the clusters 7, and negative for the clusters 2 and 3 (Table 2 and Figure 2). The anxiety sub-score of HAD also significantly and negatively correlated with cluster 3. There were no more correlations with these clusters (Supplementary Table S1).

### ROI-to-ROI analyses

Good ratios of connectivity were found for the DMN (80%), the ECN (96%), the SN (73%) and the SMN (93%).

Only one significant negative regression was observed between the Cooperativeness scores and the functional connectivity within a cluster in the DMN (*p-FDR = 0.046*) formed by the right and left middle occipital cortices, the right precuneus and the left calcarine.

## Discussion

To our knowledge, this is the first study to evaluate the relationship between personality and functional connectivity in PD. We have highlighted an association between Novelty Seeking and the functional connectivity between the nucleus accumbens and part of the orbitofrontal cortex, likely reflecting its association with the dopaminergic system. Moreover, an association between Harm Avoidance and the functional connectivity between the right temporal lobe and part of the fronto-thalamo-parietal network was revealed, highlighting its link with the serotoninergic system. Furthermore, this last functional connectivity was correlated with the anxio-depressive state of PD patients.

The first association revealed that as Novelty Seeking scores increase, connectivity between the nucleus accumbens and part of the orbitofrontal cortex increases as well. Such increased connectivity was already found in the literature in subjects with alcohol use disorder and was associated with more alcohol-seeking behavior (Bracht et al., 2021). Another study in rodents have shown that decisional impulsivity was related to the orbitofrontal cortex and nucleus accumbens activities (Wang et al., 2019). The nucleus accumbens is part of the reward system involved in emotions that may reinforce some behaviors such as impulsivity (Basar et al., 2010); while the orbitofrontal cortex, part of the prefrontal cortex, is implicated in executive functions such as inhibition (Yuan and Raz, 2014). This could explain why the modification of connectivity within these two cerebral regions may play part in impulsive behaviors (Volkow et al., 2019; Winstanley, 2007). These two regions are also related to the dopaminergic system, strongly associated with impulsivity (Baik, 2013) such as impulsive controls disorders in PD (Cormier et al., 2013; Martini et al., 2018). Therefore, their association with Novelty Seeking temperament appears highly consistent, as this personality dimension represents curious, impulsive, and extravagant traits (Cloninger et al., 1994; Hansenne, 2001). Nonetheless, this functional connectivity related to Novelty Seeking was not associated with the dose of dopaminergic treatments (levodopa equivalent dosage (LED)). The lack of correlation between Novelty Seeking scores and the LED could explain this result (Boussac et al., 2022). Hence, this association between personality and functional connectivity may be solely related to the initiation of dopaminergic treatment (and to the aforementioned change in Novelty Seeking scores during the course of PD (Boussac et al., 2021)), rather than to the dose of dopaminergic treatment.

Then, a more complex relationship was observed between Harm Avoidance and connectivity between the right temporal lobe and different clusters within the fronto-thalamo-parietal network. Correlations were either positive or negative within this network formed of the post-central gyrus (primary somatosensory areas), pre-central gyrus (primary motor areas), thalamus, parietal, and putamen. This central executive network is involved in executive functions and high-level cognitive tasks and goal-oriented tasks (Marek and Dosenbach, 2018; Spreng et al., 2010; Vendetti and Bunge, 2014), as well as the temporal lobe engaged in cognitive and emotional functions (Herlin et al., 2021). Moreover, depressive disorders are characterized by deficit in emotional and cognitive control (Zhang et al., 2022), which may explain why alterations within this fronto-thalamo-parietal network were implicated in depressive state in different studies (Lencer et al., 2018; Schultz et al., 2019; Zheng et al., 2023). Each of the cerebral regions found in our study are somehow related to depressive disorder and to the serotoninergic system in the literature: the primary somatosensory cortex (Kang et al., 2018; Wang et al., 2018), the precentral gyrus (Pawlak et al., 2022; Rolls et al., 2018), the thalamus (Brown et al., 2017), the parietal (Schultz et al., 2019), the putamen (Lu et al., 2016), and the temporal (Brown et al., 2017). Therefore, their association with Harm Avoidance temperament seems truly consistent as this personality dimension is characterized by anxiety, shyness, and fatigue (Cloninger et al., 1994; Hansenne, 2001).

Concerning cerebral networks, we have confirmed their functionality in our PD patients with high ratio of connectivity (superior to 70%) in the DMN, ECN, SN and SMN. We also discovered a negative relationship between the personality of Cooperativeness and functional connectivity within the DMN (occipital part, precuneus and calcarine sulcus): higher Cooperativeness scores were related to lower connectivity within the DMN. As the DMN is involved in self-thinking and introspection (Buckner et al., 2008), we can imagine that a modulation of its activity may be helpful to focus its attention toward more extroverted ways of thinking, which is needed to cooperate and good social interactions. Indeed, the precuneus is important for conscious information processing, through a decrease of activity, and is part of a so-called “neural correlates of consciousness” (Vogt and Laureys, 2005).

Based on our previous study comparing TCI in treated PD, in unmedicated PD and a French normative population, we have suggested that personality of PD patients may change during the course of the disease, either due to the dopaminergic treatments or the disease progression (Boussac et al., 2021).

Hence, the cerebral functional connectivity observed here in relation to personality may also evolve during the course of the disease, as it is associated to the degeneration of neurotransmitter systems in PD (Buddhala et al., 2015). For instance, reduction of dopamine levels was observed in the nucleus accumbens in PD patients, and could be related to neuropsychiatric symptoms, cognitive symptoms and impulsive-compulsive behaviors (Chang and Wang, 2023; Hammes et al., 2019; Mavridis, 2015; Mavridis et al., 2011). A decreased functional connectivity of the rostral anterior cingulate cortex with the nucleus accumbens was even associated with severity of impulsive-compulsive behavior in PD (Hammes et al., 2019), and changes in functional connectivity between the nucleus accumbens and the anterior cingulate cortex preceded apathy in PD (Morris et al., 2023). Novelty Seeking scores are usually lower in *De Novo* PD patients (Santangelo et al., 2018) and apathy is also often present in these patients (Castrioto et al., 2025; Zhang et al., 2024). Therefore, we assume that dopamine loss in the nucleus accumbens is associated with this change in temperament in PD, since functional connectivity of the nucleus accumbens was correlated here with Novelty Seeking - which may increase with the initiation of dopaminergic treatments (Boussac et al., 2021), as well as impulsive-compulsive behaviors.

A gradual loss of serotonin is also observed in PD, mainly linked to non-motor symptoms (Politis and Niccolini, 2015) such as depression (Mayeux et al., 1988). A loss of serotonin has been observed in several regions of the central executive network such as the putamen, the thalamus, the parietal and the frontal regions (Jørgensen et al., 2021; Politis and Niccolini, 2015). We therefore hypothesize that the generally higher scores of Harm Avoidance observed in PD patients (Boussac et al., 2021; Santangelo et al., 2018) are related to this serotoninergic deficit in the central executive network.

Some limitations would need to be addressed in further studies. First, the sample size could be enlarged to ensure our results would be replicable to a larger population. Hence, some co-variables could be added in the imaging analyses within a larger cohort. Also, more clinical variables related to impulsivity, for example, should be studied to evaluate their association with the functional connectivity found associated with Novelty Seeking scores, to enhance the interpretations of our results. Finally, a similar study in healthy controls would be interesting to see if our results are specific to PD patients or could be generalized to a larger extent. In the same idea, a longitudinal study evaluating functional connectivity and personality through the course of PD could be useful to truly estimate the relationship between the neurodegenerative process in PD and the changes of personality.

In conclusion, certain TCI personality dimensions appear to be meaningfully associated with neuro-functional correlates, supporting some of Cloninger’s hypotheses in PD: more specifically, the association of Novelty Seeking with the dopaminergic system and Harm Avoidance with the serotoninergic system. At present, these results should only be considered in PD patients. Hence, these associations may help explain how personality changes can occur during PD, in parallel with the ongoing neurodegenerative processes.

## Data Availability

The raw data supporting the conclusions of this article will be made available by the authors, without undue reservation.
